# APOE4 carriers resistant to cognitive decline show unique relationships between cerebrovascular response to exercise and dual-task cognitive-balance performance

**DOI:** 10.3389/fnins.2026.1716713

**Published:** 2026-04-13

**Authors:** Jacqueline A. Palmer, Sandra A. Billinger

**Affiliations:** 1 Division of Physical Therapy and Rehabilitation Science, Medical School, University of Minnesota, Minneapolis, MN, United States; 2 Department of Neurology, School of Medicine, University of Kansas Medical Center, Kansas City, KS, United States; 3 University of Kansas Alzheimer's Disease Research Center, Fairway, KS, United States; 4 Department of Molecular & Integrative Physiology, University of Kansas Medical Center, Kansas City, KS, United States

**Keywords:** aging, *Apolipoprotein E4*, brain health, brain resilience, cerebral blood flow, cognition, response inhibition, transcranial Doppler ultrasound

## Abstract

**Background:**

Cognitive-motor dual-tasking is an early marker for cognitive impairment, with particular implications for *Apolipoprotein E4 (APOE4)* carriers who are at higher genetic risk for Alzheimer's disease. While *APOE4* carriers typically show accelerated cognitive decline and impaired cerebrovascular function with aging, exceptions to this norm exist and may provide insights into resilience mechanisms. The relationship between cerebrovascular response and cognitive-motor dual-task performance in cognitively-normal *APOE4* carriers who maintain preserved function remains unclear.

**Methods:**

Thirty cognitively-normal older adults (76 ± 4 years, 8 *APOE4* carriers, 22 non-carriers) completed clinical balance and cognitive testing under single-task and dual-task conditions. Balance performance was assessed as distance traversed during challenging beam walking. Cognitive performance was assessed as response time during an auditory Stroop test. Transcranial Doppler ultrasound measured cerebrovascular response to moderate-intensity aerobic exercise. We tested group differences in cognitive-balance dual task performance and relationships between cerebrovascular response and dual-task interference (DTI) in balance and cognitive domains, and effects of *APOE4* genotype on these relationships.

**Results:**

No differences in cerebrovascular response or dual-task performance were observed between *APOE4* carriers and non-carriers. However, *APOE4* carriers displayed unique cerebrovascular-behavioral relationships. In *APOE4* carriers, higher cerebrovascular response to exercise was associated with less balance DTI (r = 0.839, *p* = 0.009) and less cognitive DTI (r = 0.832, *p* = 0.020), while no relationships were observed in non-carriers (*p* > 0.187).

**Conclusions:**

Cognitively-normal *APOE4* carriers with preserved cognitive-balance dual-task function represent exceptions that may model aging resilience mechanisms. The unique cerebrovascular-behavioral relationships suggest that maintaining cerebrovascular function supports neuromotor and neurocognitive resilience to dual-task challenges in genetically vulnerable populations.

## 1 Introduction

Approximately 25% of the U.S. population carry at least a single *Apolipoprotein E4* (*APOE4*) allele, the strongest known genetic risk factor for Alzheimer's disease (AD) (Heffernan et al., 2016) and a high risk for other diseases involving cardiovascular health (e.g., heart attack and stroke) (Haan and Mayeda, 2010). Increasing evidence points to an early and key role of cerebrovascular dysfunction in the pathogenesis of Alzheimer's disease (Iturria-Medina et al., 2016; Wolters et al., 2017; Sweeney et al., 2018). The *APOE4* genotype has been the most commonly studied genetic variant linked to brain function and appears to act synergistically with cardiovascular health (e.g., blood pressure, lipid profiles, white matter hyperintensities) to influence cognitive decline (Haan and Mayeda, 2010; Oberlin et al., 2015; Bender and Raz, 2012; de Leeuw et al., 2004; Zade et al., 2010). When brain vascular function is attenuated, this can reduce or slow down the clearance of amyloid-beta, a neurobiological hallmark for AD- and promote its accumulation in the brain (Solis et al., 2020). For *APOE4* carriers, who show accelerated amyloid-beta pathology (Liu et al., 2017), maximizing cerebrovascular function may be critical for maintenance of brain health with aging. For example, cerebrovascular function can be characterized as responsivity under conditions of *physiologic stress* (e.g., sit-to-stand positional changes, aerobic exercise, heat stress, hypoxia) (Steinback and Poulin, 2016; Ogoh and Ainslie, 2009a,b; Ogoh et al., 2013; Sato et al., 2011; Sisante et al., 2019; Palmer et al., 2025) that can play an important role in maintaining brain metabolism and function with aging (Bundo et al., 2002), as the damaging effects of repeated transient disruption of blood, glucose, and oxygen supply to brain tissue accumulate over time (Tarumi and Zhang, 2018). Recent studies involving exercise interventions suggest that the protective effects of exercise on risk for future dementia appear to be even stronger in individuals who carry *APOE4* (Smith et al., 2013; Kaufman et al., 2021a). Supporting this notion, our group previously showed that, despite having higher amyloid-beta deposition compared to non-carriers, cognitively-normal older adult *APOE4* carriers who maintained exceptionally high cerebrovascular response to a bout of aerobic exercise showed no difference in cognitive executive function performance (Palmer et al., 2022a). This is remarkable because of the high genetic vulnerability of *APOE4* carriers to early signs of AD and the fact that impaired cognitive executive function is one of the earliest cognitive manifestations of mild cognitive impairment (MCI) that can progress to dementia (Hutchison et al., 2010; Kirova et al., 2015).

Decline in balance and gait function may occur several years before individuals meet clinical diagnosis for mild cognitive impairment (MCI) (Rosano and Snitz, 2018; Quan et al., 2017; Hoogendijk et al., 2020), suggesting that motor behavior may be a more sensitive indicator of underlying neuropathology that precipitates clinical cognitive syndrome. The emergence of cognitive interference in balance and walking over the course of aging is one of the most prevalent clinical phenomenon that emerges with aging (Lundin-Olsson et al., 1997; Duckrow, 1999). A person's ability to perform cognitive-motor dual-tasking may reflect individual neural capacity (Tombu and Jolicoeur, 2005, 2002, 2003; Palmer et al., 2021) that supports neurocognitive and neuromotor resilience imposed by competing attentional demand (Montine et al., 2019). Clinical dual-task paradigms can be used to assess cognitive-motor interference, in which the individual is asked to simultaneously perform a cognitive task while balancing/walking and the change in their performance in either or both tasks is measured (Whitson et al., 2018; Plummer and Eskes, 2015). Specifically, greater degradation of *balance and gait* performance under cognitive loading [i.e., greater dual-task interference (DTI)] is an early and sensitive indicator of behavioral dysfunction in older adults (Lundin-Olsson et al., 1997; Montero-Odasso et al., 2012; Brown et al., 1999; Leone et al., 2017; Morris et al., 2016; Rankin et al., 2000; Woollacott and Shumway-Cook, 2002), and can predict future dementia (Montero-Odasso et al., 2017) and falls (Montero-Odasso et al., 2012; Shumway-Cook et al., 1997). One pilot study showed that older adult *APOE4* carriers showed greater cognitive DTI during walking, in which the cognitive task domain of executive function showed an even greater effect compared to a working memory task in *APOE4* carriers during dual-task gait (Whitson et al., 2018). Recently, our group showed that there is a relationship between higher cerebral blood velocity and cognitive-balance dual-task behavior in cognitively-normal older adults, especially with advanced age (Palmer et al., 2024). The effect of advanced age is notable because preserved cognitive function becomes more meaningful with age, as the magnitude of average decline in cognitive performance over time is disproportionately influenced by age, and manifestations of genetic risk becomes critical factors for disease risk (Rogalski, 2019; Burke et al., 2019). This poses the question of whether modifiable factors such as brain vascular health contribute to the early clinical manifestations of cognitive-motor dual-task interference that could be therefore be targeted and modified with clinical intervention during preclinical disease stages in highly vulnerable older adults who carry *APOE4*.

Our group previously showed that, despite having greater amyloid-beta deposition, cognitively-normal *APOE4* carriers with more robust cerebrovascular response to aerobic exercise had higher cognitive response inhibition performance under single-task conditions (Palmer et al., 2022a). Cerebrovascular response to physiologic stress like aerobic exercise, measurable through clinically feasible and cost-effective transcranial Doppler ultrasound (TCD) (Sisante et al., 2019; Billinger et al., 2017; Ward et al., 2018; Palmer et al., 2022b), serves as a more sensitive early indicator of cognitive dysfunction than resting assessments, which fail to detect subtle vascular impairments in older adults (Sisante et al., 2019; Xie et al., 2016). Based on our previous findings that cerebrovascular response to exercise supports single-task cognitive function in cognitively-normal APOE4 carriers despite elevated amyloid-beta (Palmer et al., 2022a), we hypothesized that cerebrovascular function may serve as a resilience mechanism extending beyond single-task conditions. Specifically, we hypothesized that cognitively-normal *APOE4* carriers in advanced age who maintain preserved cerebrovascular response to exercise would show unique cerebrovascular-behavioral relationships in dual-task performance compared to non-carriers. We reasoned that dual-task conditions, which impose greater cortical resource demands through competing attentional loads (Tombu and Jolicoeur, 2005, 2002, 2003; Palmer et al., 2021) would reveal cerebrovascular health as a critical mechanism supporting neuromotor and neurocognitive resilience in this genetically vulnerable population. To test this hypothesis, we assessed whether cognitively-normal *APOE4* carriers in advanced age (70+ years) differed from non-carriers in single- and dual-task cognitive-balance performance, and whether cerebrovascular response to exercise differentially predicted dual-task interference as a function of *APOE4* genotype.

## 2 Materials and methods

### 2.1 Participants

Thirty participants (76 ± 4 years, 19 females) from the University of Kansas Alzheimer's Disease Research Center (P30AG072973) (Iturria-Medina et al., 2016; Solis et al., 2020; Liu et al., 2017; Steinback and Poulin, 2016) were selected for this study (Table 1). Participants were recruited from the ADRC cohort based on having previously completed a vascular assessment visit with our laboratory, during which viable transcranial Doppler ultrasound signals were confirmed (Sisante et al., 2019). For inclusion in this analysis, participants had to be (Heffernan et al., 2016) age 70–90 years (Haan and Mayeda, 2010), have normal cognition (see below) (Iturria-Medina et al., 2016), have absence of neurologic or orthopedic disability to prevent independent standing and walking, and (Wolters et al., 2017) speak the English language. Exclusion criteria were (Heffernan et al., 2016) insulin-dependent diabetes (Haan and Mayeda, 2010), peripheral neuropathy affecting somatosensation (Iturria-Medina et al., 2016), active coronary artery disease and congestive heart failure. The University of Kansas Institutional Review Board approved this protocol (IRB#: STUDY 00147888) and all participants provided written informed consent.

**Table 1:** Participant characteristics.

Metric	ALL (*n* = 30)	APOE4 E4/E3 (*n* = 8)	Non-carriers E3/E3 (*n* = 22)	*P* value
Age	76 ± 4	75 ± 4	76 ± 5	*p =* 0.584
Sex (F/M)^∞^	19/11	3/5	16/6	*p =* 0.077
Resting mean arterial pressure (mmHg)	93 ± 22	96 ± 18	91 ± 24	0.585
Resting Heart rate (bpm)	73 ± 9	71 ± 11	74 ± 8	0.152
Exercise heart rate (bpm)	107 ±7	103 ± 10	109 ± 5	*p =* 0.056
CBFv response to exercise (Δ)	1.6 ± 4.4	1.5 ± 3.84	1.65 ± 4.73	*p =* 0.943
MAP response to exercise (Δ)	11 ± 12	10 ± 14	11 ± 12	*p =* 0.952
Exercise Watts	50 ± 23	58 ± 32	47 ± 19	*p =* 0.266

Values are depicted as mean ± SD. ^*^p < 0.05; ^∞^self-reported.

### 2.2 Clinical screening for cognitive impairment and eligibility

Participants completed extensive neuropsychological testing and Clinical Dementia Rating (CDR) assessment through the University of Kansas Alzheimer's Disease Research Center, administered by a qualified clinician and psychometrist. Only individuals with normal cognitive status (CDR = 0) and *APOE* genotyping were included in this analysis.

### 2.3 Behavioral assessments of balance and cognitive performance

Participants completed a challenging beam walking task (Uematsu et al., 2018; Sawers and Ting, 2015; Kemp et al., 2018) in which they walked across a 16-foot long narrow beam (3.5-inch width,1-inch height) (Uematsu et al., 2018). This beam walking task can reliably detect dynamic balance proficiency across older adults with high and low balance function (Sawers and Ting, 2015), can detect age-related differences in cognitive-balance dual-task interference (Uematsu et al., 2018), and may be more sensitive in detecting impairment compared to conventional clinical tests (Hortobágyi et al., 2019). Older adults who achieved perfect beam scores on the first two trials were progressed to a narrower beam width (1-inch), starting on the narrow beam with a baseline score of 16 feet.

#### 2.3.1 Single-task balance performance

**Figure 1:**
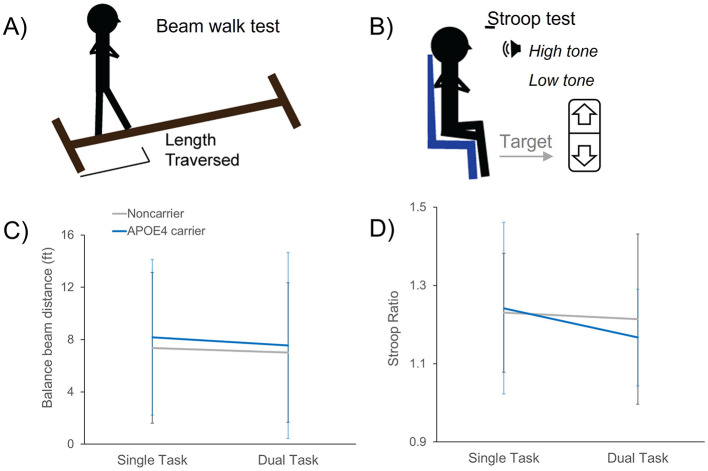
Balance and cognitive performance during single- and dual-task conditions in APOE4 carriers and noncarriers. Paradigms for assessing balance and cognitive performance across all older adults showing the beam walk task **(A)** and the Stroop response inhibition task **(B)**. There were no group differences between APOE4 carriers and noncarriers on the beam walk distance traversed performance **(C)** or in cognitive Stroop task performance **(D)** during either single- or dual-task conditions. Data shown as mean ± standard deviation.

Participants wore a safety belt and were instructed to fold their arms across their chest, fix their gaze straight ahead at a point on the wall straight ahead at eye level, and walk forward across the beam at a their preferred speed without stepping off the beam or uncrossing their arms. A trial was stopped when the participant stepped off the beam, walked sideways, or unfolded their arms, and their foot placement position was marked (Figure 1A).

#### 2.3.2 Dual-task balance performance

Following the single-task condition, participants completed a dual-task beam walk during simultaneous performance of a cognitive task (Uematsu et al., 2018). The secondary cognitive task required the participant to verbally count backward by 3′s starting at a random integer number between 20 and 100 verbally stated by the experimenter immediately following the cue to begin the beam walk. The mean beam distance traversed and gait speed across 2 trials for each single-task and dual-task balance conditions was used to compute balance DTI (below).

#### 2.3.3 Single-task cognitive performance

To enable more precise quantification of cognitive performance, participants completed a cognitive response inhibition task using the auditory Stroop test delivered through E-Prime Software (Pittsburgh, PA). The Stroop test is a widely used cognitive executive function test assessing the ability to inhibit the prepotent and undesired response (Hutchison et al., 2010; Stroop, 1935). A 50-stimulus sequence of auditory words stating either “high” or “low” were emitted at 0.5 Hz in either high or low tones, with half of the word-tone stimuli being congruent (e.g., “high” in a high tone) and half being incongruent (e.g., “high” in a low tone). Participants were asked to respond to the tone of the sound and ignore the meaning of the word in a 50-stimuli sequence delivered at 0.5 Hz by clicking the up (high tone) or down (low tone) button on a hand-held clicker as quickly as possible while maintaining accuracy (Figure 1B). Participants completed a 10-stimuli practice trial prior to the 50-stimuli test sequence.

#### 2.3.4 Dual-task cognitive performance

Following the single-task condition, participants completed the auditory Stroop test while simultaneously standing on an unstable balance board (Fluidstance Level Balance Board) (Santa Barbara, CA). The level of balance challenge was individually adjusted with the addition or removal of a convex board cap based on the participant's balance ability, determined by the participant's level of stability while standing on the board assessed by a licensed physical therapist. Participants were required to fold their arms across their chest and affix their gaze at an eye-level point on the wall while holding the remote clicker.

Stroop test accuracy was >90% for each task condition (single-task: 95% ± 6%; dual-task: 91% ± 15%), and all incorrect trials were removed from response time analyses. A Stroop ratio was computed as the mean response times of all trials with congruent stimuli over the incongruent stimuli. The Stroop ratio during each the single- and dual-task conditions was used to compute *dual-task interference (DTI)* as:


$$\begin{array}{c} DTI=\frac{ST\;performance-DT\;performance}{ST\;performance}*100\% \end{array}$$

For balance DTI, the value was multiplied by (−1), such that a negative DTI value for each balance and cognition indicates worsening of performance (i.e., slower response times during incongruent relative to congruent stimuli) between the single- and dual-task conditions (Plummer and Eskes, 2015).

### 2.4 Cerebrovascular assessment during aerobic exercise

Briefly, participants arrived to the laboratory (22–24 degrees Celsius) in the morning and abstained from caffeine for 12 h, intense exercise for 24 h, and a large meal for 2 h. TCD was used to assess cerebral artery blood velocity (CBFv) during a single bout of moderate-intensity aerobic exercise (Billinger et al., 2017; Ward et al., 2018). A 2-MHz TCD probe (RobotoC2MD, Multigon Industries) was placed over the left temporal window. The left anterior cerebral artery (ACA) was targeted because of its vascular supply to lower extremity sensorimotor cortical regions involved in balance and walking behaviors involved in the present study. The left MCA was used if the ACA signal was absent, and the right side was used if the signal was absent on the left side. A 5-lead electrocardiogram (ECG) (Cardiocard, Nasiff Associates, Central Square, New York) continuously monitored and recorded heart rhythm. Continuous beat-to-beat mean arterial pressure (MAP) was recorded through a cuff around the left middle finger (Finapres Medical Systems, Amsterdam, the Netherlands). Following TCD, MAP, and ECG setup, we implemented a moderate-intensity aerobic exercise protocol on a recumbent stepper (NuStep T5XR) (Billinger et al., 2017; Ward et al., 2018). Prior to data recording, participants familiarized themselves with the reciprocal stepping motion at a cadence of 100 steps per minute. We defined our moderate-intensity target as 45%−55% of heart rate (HR) reserve using the Karvonen formula. We conducted an individualized calibration procedure to establish each participant's optimal work resistance: beginning with the stepper set to 30 watts, we added 10 watts at regular intervals while monitoring HR until participants achieved and maintained their prescribed HR reserve zone of 45%−55%. After establishing this personalized resistance level, we halted the calibration exercise and allowed participants to recover quietly for no less than 10 min, ensuring complete physiological stabilization before commencing the experimental trial. The experimental recording consisted of 90 seconds of seated rest on the recumbent stepper, followed by 6 min of continuous moderate-intensity exercise. We employed a graduated exercise initiation protocol to minimize potential Valsalva maneuvers and prevent abrupt physiological fluctuations: participants began stepping at 60% of their individualized target wattage and progressively increased resistance at 10-second intervals, achieving their full target workload 30 seconds after exercise initiation. Participants then maintained this moderate-intensity workload for the remaining duration of the 6-min exercise period. Custom MATLAB software (The Mathworks Inc.) using an analog-to-digital data acquisition unit (NI-USB-6212, National Instruments) acquired MCAv (500 Hz), synchronized across the cardiac cycle (Billinger et al., 2017; Ward et al., 2018). Data were visually inspected and discarded when R-to-R intervals were >5 Hz or changes in peak CBFv exceeded 10 cm/s in a single cardiac cycle. Trials with < 85% samples were discarded. Mean CBFv was calculated from the area under the curve for each cardiac cycle and analyses were conducted using 3-s time-binned mean values over the entire rest and exercise period, as described previously (Ward et al., 2018). We calculated cerebrovascular response (CVR) and mean arterial pressure (MAP) response to exercise as the difference between mean CBFv and mean MAP during minutes 3 to 4.5 at exercise steady state and mean CBFv during the rest prior to the start of exercise.

### 2.5 *APOE* genotyping

Participants provided whole blood samples that were drawn and stored frozen at −80 degrees Celsius. Genetic analyses was performed using a Taqman single nucleotide polymorphism (SNP) allelic discrimination assay (ThermoFisher) to determine *APOE* genotype. *APOE4, APOE3*, and *APOE2* alleles were determined using Taqman probes to the two *APOE*-defining SNPs, rs429358 (C_3084793_20) and rs7412 (C_904973_10) (Kaufman et al., 2021b; Vidoni et al., 2021). Individuals were classified as *APOE4* carrier in the presence of 1 or 2 *APOE4* alleles (e.g., E3/E4, E4/E4). Individuals with homozygous E3 (e.g., E3/E3) were classified as a non-carriers. We excluded any individual who carried one or two copies of *APOE2*, as *APOE2* is associated with reduced risk for AD and could affect cerebrovascular and behavioral results of the present study (Kaufman et al., 2021a; Whitson et al., 2018). All assessors were blinded to the participant's genotype.

### 2.6 Statistical analyses

We confirmed normality and heterogeneity of variance using Kolmogorov-Smirnov and Levene's tests, respectively. We used two-way mixed analysis of variance (ANOVA) tests to assess balance beam performance and cognitive Stroop task performance between single- and dual-task conditions within each participant and between *APOE4* carriers and non-carriers. Given the small and unbalanced sample size, we replaced Pearson's *r* with Bootstrapped Pearson's correlation coefficients (5,000 samples, Bias Corrected Accelerated 95% Confidence Intervals) to test the relationship between CBFv response to exercise and DTI measures within each *APOE4* genotype group, providing a robust, nonparametric estimate of the confidence interval. Given the effect of *APOE4* on AD risk and the link between DTI and AD in older adults (Osoba et al., 2019; Alshammari et al., 2018; Freedman et al., 2014), we then used two-way multiple linear regression (MLR) analyses (factors: APOE genotype, CBFv response to exercise, genotype-by-CBFv response to exercise) to test whether cerebrovascular-behavioral relationships differed as a function of *APOE4*. MLR models testing the genotype-by-CBFv response interaction were validated using bootstrapping (5,000 samples, BCa Confidence Intervals) to ensure the robustness of the results given the small, unbalanced sample size. No formal a priori power analysis was conducted for this study. Sample size was determined by feasibility constraints, as participants were selected based on availability within the ADRC cohort and successful identification of viable transcranial Doppler signals during prior vascular assessments. Given the preliminary nature of this investigation and the technical limitations of TCD signal acquisition, we employed robust statistical methods including bootstrapped correlation coefficients and confidence intervals (5,000 samples, BCa 95% CI) to provide reliable effect estimates despite the modest and unbalanced sample size. All statistical tests were based on a priori, directional hypotheses derived from established literature, and therefore, no formal correction for multiple comparisons (e.g., Bonferroni) was applied. Effect estimates are presented with their BCa 95% Confidence Intervals as a robust measure of uncertainty, which is the primary metric for statistical inference in the current study. All analyses were performed using SPSS version 29 with an *a priori* level of significance set to 0.05.

## 3 Results

Two participants had unstable TCD signals with excessive noise during aerobic exercise; these participants were discarded from subsequent analysis. One participant had an ACA signal on the right side only; the right side was used for this participant. The ACA signal could be located and stabilized in 21/30 participants; for the remaining 9 participants, the left MCA signal was used for analyses. Two out of these 9 participants only possessed TCD signals on the right side, and thus the right side was used. Two participants (n = 1 *APOE4* carrier, n = 1 non-carrier) did not follow instructions for the Stroop test during single and/or dual-task conditions; these participants were discarded from cognitive analyses. Stroop test accuracy on the single-task was 95% ± 6% and 91% ± 15% under the dual-task condition. No differences in demographics of age or sex were observed between participants in the *APOE4* and non-carrier groups (*p* > 0.05). There were no differences in cardiovascular or exercise metrics between groups, including baseline blood pressure, mean arterial pressure response to exercise, heart rates, CBFv response to exercise, or exercise wattage (see Table 1).

### 3.1 Balance and cognitive performance under single and dual-task conditions in *APOE4* carriers compared to non-carriers

Cognitively-normal older adults with *APOE4* showed no differences in balance or cognitive performance compared to non-carriers, regardless of task condition. When comparing balance beam performance and cognitive Stroop task performance between single- and dual-task conditions as a function of *APOE4* genotype, there were no effects of condition or group. There were no group-by-condition interaction effects for metrics of balance beam distance traversed, balance beam gait speed, or cognitive Stroop ratio task performance (*F*_1, 28_ ≤ 0.93, *p* ≥ 0.343) and no main effects of condition (*F*_1, 28_ ≤ 2.57, *p* ≥ 0.120) or group (*F*_1, 28_ ≤ 0.71, *p* ≥ 0.406) (Figures 1C, D).

### 3.2 Effect of *APOE4* genotype on the relationship between cerebrovascular response to exercise and dual-task balance and cognitive performance

**Figure 2:**
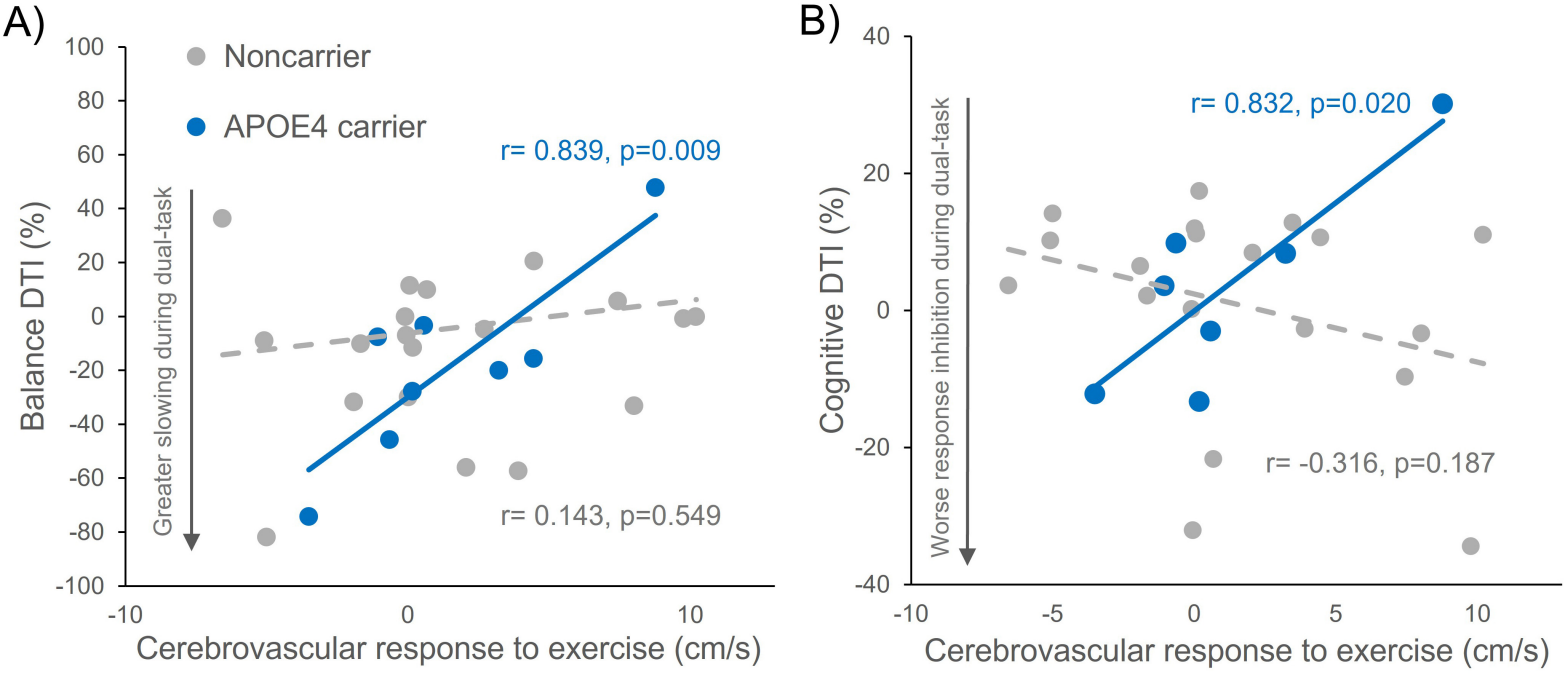
Relationship between cerebral blood velocity (CBFv) response to aerobic exercise and dual-task interference (DTI) in APOE4 carriers and non-carriers in each domain of balance **(A)** and cognitive performance **(B)**. There was a significant relationship in APOE4 carriers in each the balance and cognitive domains of dual-task interference, in which older adults with APOE4 who displayed higher cerebrovascular responses exhibited less balance DTI in slowing of gait speed during the beam walk task (r = 0.839, *p* = 0.009) **(A)** and less cognitive DTI in worsening of response inhibition performance (r = 0.832, *p* = 0.020) **(B)**, while noncarriers showed no relationships.

Older adults with *APOE4* displayed stronger cerebrovascular-behavioral relationships across balance and cognitive domains of dual-task interference compared to non-carriers. Within the balance domain, *APOE4* carriers exhibited a positive relationship between higher cerebrovascular response to exercise and less balance DTI (lesser decline in gait speed during beam walking, *r* = 0.839, *p* = 0.009). To confirm the robustness of this finding given the small sample size, we performed bootstrapping analyses, which revealed a Bias Corrected Accelerated (BCa) 95% Confidence Interval that excluded zero (95% CI: [0.066, 0.976]). No significant relationship was observed in non-carriers (*r* = 0.143, *p* = 0.549; 95% CI: [−0.344, 0.529]) (Figure 2A). A trend for a similar relationship in *APOE4* carriers was also observed for balance DTI on beam distance traversed in *APOE4* carriers (*r* = 0.613, *p* = 0.106; 95% CI: [−0.549, 0.925]) and showed no relationship in non-carriers (*r* = 0.298, *p* = 0.202; 95% CI: [−0.077, 0.599]), but failed to meet our adopted level of significance with a CI containing zero. Within the cognitive domain, *APOE4* carriers with a greater cerebrovascular response to exercise exhibited lesser decline in cognitive Stroop task performance from single- to dual-task conditions (*r* = 0.832, *p* = 0.020; 95% CI: [0.014, 0.982]) while no relationship was observed in the non-carriers (*r* = −0.316, *p* = 0.187; 95% CI: [−0.703, 0.132]) (Figure 2B).

We performed exploratory analyses to test whether the observed cerebrovascular-behavioral relationships significantly differed between *APOE4* carriers and non-carriers. In the domain of cognitive performance, there was a significant interactive effect of *APOE4* genotype with cerebrovascular response to exercise on cognitive Stroop DTI (model: *F*_3, 25_ = 2.42, *p* = 0.093, *R*^2^ = 0.248, adjusted *R*^2^ = 0.146; interaction: *t* = 2.634, *p* = 0.015), in which the relationship between higher cerebrovascular response to exercise and more positive Stroop DTI in *APOE4* carriers was stronger than the relationship in non-carriers (Figure 2B). The BCa 95% Confidence Interval for the interaction term was [2.103, 5.218], which does not contain zero, thereby supporting the relationship between CBFv and cognitive dual-task performance differs as a function of *APOE4* genotype. In the domain of balance performance, the genotype-by-cerebrovascular response to exercise interaction effect was not statistically significant (Balance beam distance traversed: model: *F*_3, 27_ = 2.414, *p* = 0.091, *R*^2^ = 0.232, adjusted *R*^2^ = 0.136; interaction: *t* = 1.639, *p* = 0.114, 95% CI: [−16.35, 18.81]; Balance beam gait speed: model: *F*_3, 27_ = 1.45, *p* = 0.253, *R*^2^ = 0.153, adjusted *R*^2^ = 0.048; interaction: *t* = 1.30, *p* = 0.208; 95% CI: [−0.826, 10.063] (Figure 2A).

To examine whether these relationships were specific to cerebrovascular response rather than reflecting global hemodynamic changes during exercise, we conducted exploratory Pearson's correlation analyses between MAP response to exercise and balance DTI and cognitive DTI within each genotype group. No significant relationships were observed in *APOE4* carriers or non-carriers (all *p* > 0.05), suggesting that the unique cerebrovascular-behavioral associations reported above are not attributable to systemic blood pressure responses to exercise.

## 4 Discussion

Our study provides initial results for *APOE4* genotype as a biomarker of differential vulnerability, specifically identifying individuals in whom cerebrovascular function plays a heightened role in supporting cognitive-motor performance. Cognitively-normal *APOE4* carriers in advanced age who maintain preserved cerebrovascular function represent exceptions that may reveal aging resilience mechanisms. Rather than exhibiting the expected decline in cerebrovascular health and cognitive-motor performance typical of *APOE4* carriers of this age, this subgroup maintained function comparable to non-carriers while displaying unique cerebrovascular-behavioral relationships. This pattern of preserved function paired with differential relationships with specific biological systems is consistent with theoretical frameworks of brain resilience and compensation (Montine et al., 2019). Here, the well-preserved cognitive-motor dual-task behavior and cerebrovascular function paired with unique cerebrovascular-behavioral relationships in this subgroup of cognitively-normal *APOE4* carriers in advanced age provides preliminary findings for larger mechanistic studies and clinical trials to further elucidate brain resilience mechanisms in this unique aging phenotype.

Despite having genetically higher risk for the development of AD and advanced age, the *APOE4* carriers in the present study were cognitively-normal and showed no differences in cognitive or balance behavior or cardio- or cerebrovascular metrics assessed here (Table 1), suggesting functional resilience to expected decline. While previous studies show that *APOE4* carriers on average display lower markers of vascular health compared to non-carriers (Haan and Mayeda, 2010; Oberlin et al., 2015; Bender and Raz, 2012; de Leeuw et al., 2004; Zade et al., 2010), the preserved cerebrovascular response to aerobic exercise in our cohort may implicate protective, modifiable factors such as physical activity in genetic AD risk. The absence of dual-task deficits in our cohort of *APOE4* carriers is particularly noteworthy given that our cognitively-normal older adults were 10 years older on average (75 years) compared to Whitson et al.'s cohort (65 years) (Whitson et al., 2018), which demonstrated cognitive performance deficits during dual-task conditions in *APOE4* carriers. This preserved function at advanced age is remarkable because cognitive response inhibition—the ability to suppress an undesired default or automatic response in the presence of interfering stimuli—has been identified as an early marker of impaired cognition that can distinguish between older adults who are cognitively normal compared to those diagnosed with mild cognitive impairment (Hutchison et al., 2010). The maintained cerebrovascular-behavioral function in this older subgroup of *APOE4* carriers suggests exceptional resilience mechanisms that may be supported by higher cerebrovascular health.

Existing evidence shows that brain resilience, the ability to cope with challenge, declines with age and influences the susceptibility to dementia (Montine et al., 2019). Dual-task performance may be particularly sensitive for probing resilience because it challenges neural capacity. When individuals must simultaneously allocate cognitive resources to balance control and response inhibition, the limits of their neural capacity become apparent (Tombu and Jolicoeur, 2005, 2002, 2003). Preserved dual-task function becomes increasingly rare and meaningful with advancing age (Rogalski, 2019), as genetic risk factors exert stronger influence on individual phenotype (Rogalski, 2019; Burke et al., 2019). Thus, cognitively-normal *APOE4* carriers in their mid-70s who maintain dual-task performance comparable to non-carriers may represent a resilience phenotype. The absence of group differences in cerebrovascular function, despite *APOE4* genotype typically associating with vascular impairment, may implicate protective, modifiable factors such as physical activity, whose multifaceted effects (Eggenberger et al., 2016) could support both cerebrovascular response and cognitive-motor performance in this subgroup with high genetic vulnerability.

The unique cerebrovascular-behavioral relationships in *APOE4* carriers may be explained by biological mechanisms that reflect neural and vascular compensation that emerge in the presence of genetic vulnerability. Dual-task conditions impose greater cortical resource demands through competing attentional loads (Tombu and Jolicoeur, 2005, 2002, 2003), and when neural capacity is challenged, adequate cerebrovascular function may become critical for maintaining performance. This dependency may be heightened in *APOE4* carriers, who face greater vulnerability to vascular and metabolic dysfunction (Haan and Mayeda, 2010; Solis et al., 2020). The compensatory interpretation aligns with our recent findings in a larger cohort showing that cognitively-normal *APOE4* carriers display anticipatory cerebrovascular responses prior to movement initiation; these responses were absent in *APOE4* carriers with MCI or AD, suggesting that active cerebrovascular compensation supports maintained function in resilient individuals but may be lost with disease progression (Palmer et al., 2025). This pattern suggests that active cerebrovascular compensation supports maintained function in resilient individuals but may be lost with disease progression. Similarly, the cerebrovascular-behavioral relationships observed here may represent compensatory reliance on vascular health to support cognitive-motor performance under demanding dual-task conditions. Importantly, that study also demonstrated that peripheral MAP responses during orthostasis did not differ in magnitude between *APOE4* carriers and non-carriers, and that cerebral blood velocity responses were genotype-specific (Palmer et al., 2025). This cerebrovascular specificity is consistent with the null relationships between MAP response to exercise and dual-task interference observed in the present study, and together these findings across two distinct hemodynamic challenges suggest that *APOE4*-related vascular differences are cerebral rather than systemic in origin. Both othostatic and aerobic exercise paradigms share the feature of hemodynamic challenge as the condition necessary to expose these genotype-specific cerebrovascular differences, further supporting that resting cerebrovascular measures may be insufficient or insensitive to detect *APOE4*-specific effects in cognitively normal older adults. Given that the compensatory responses identified in our prior work were present only in cognitively-normal *APOE4* carriers, the cerebrovascular-behavioral relationships observed in the present study may reflect an active compensatory phase that is subsequently lost as carriers progress toward clinical syndrome. This mechanistic framework supports the vascular hypothesis of AD, which posits that cerebrovascular dysfunction precedes neurodegenerative processes and contributes to inflammatory cascades that precipitate disease (Solis et al., 2020; Inzitari et al., 2013). Our findings extend this hypothesis by suggesting that maintained cerebrovascular function may serve as a modifiable resilience mechanism warranting future investigation to determine whether it contributes to delay or prevention of the cognitive-motor decline typical of the *APOE4* genotype.

These mechanistic insights point toward precision-based approaches for maintaining cognitive-motor function in aging. If cerebrovascular health serves as a compensatory mechanism supporting dual-task performance in genetically vulnerable populations, interventions targeting vascular function may be preferentially effective in *APOE4* carriers. Aerobic exercise interventions, which benefit cerebrovascular function more for *APOE4* carriers than non-carriers (Smith et al., 2013; Kaufman et al., 2021a), may represent a particularly promising modifiable target. The dual-task paradigm itself may have clinical utility beyond research contexts. Cognitive-balance dual-tasking challenges neural capacity (Tombu and Jolicoeur, 2005, 2002, 2003) and may serve as a behavioral probe for identifying older adults whose performance relies heavily on cerebrovascular support, potentially indicating those who would benefit most from vascular-targeted interventions. Building upon our work showing cerebrovascular response supports single-task cognitive function in *APOE4* carriers (Palmer et al., 2022a) and results from the MOBILIZE Boston study linking cerebrovascular health to walking function in older adults (Sorond et al., 2011, 2010; Jor'dan et al., 2017), dual-task assessment may offer an accessible clinical tool for detecting individuals at risk for cognitive-motor decline who could benefit from early intervention. Future clinical trials may test whether exercise interventions that enhance cerebrovascular response can prevent or delay dual-task interference in higher-risk older adults, using *APOE4* genotype to stratify treatment response. Such precision-based approaches may maximize intervention effectiveness by targeting modifiable mechanisms (cerebrovascular health) in populations with greatest vulnerability and potential for benefit (*APOE4* carriers showing early signs of dual-task decline).

### 4.1 Strengths and limitations

Subjecting participants to a comprehensive cognitive battery enabled us to confidently capture cognitively-normal older adults in advanced aging, who may be underrepresented in cognitively-normal cohorts given the pivotal role age plays in cognitive decline (Rogalski, 2019). Nevertheless, the modest sample size and cross-sectional nature identifies areas for further investigation by larger investigations with longitudinal measures. The unbalanced sample size (APOE4 carriers *n* = 8, non-carriers *n* = 22) limits the statistical power of the mixed ANOVA to detect group × condition interactions should they exist; findings from this analysis should be interpreted in the context of this constraint. While there were no differences between age and sex between *APOE4* carriers and non-carriers, age, and sex affect cerebrovascular-behavioral relationships (Palmer et al., 2024, 2022b) and warrants further investigation in larger studies. Transcranial Doppler ultrasound offers clinical applicability, but it offers limited regional specificity compared to MR-based techniques (Palmer et al., 2022c). The focus on a single cognitive domain across balance dual-task conditions restricts interpretation to cognitive response inhibition contexts. Variations may arise when applying this paradigm to other cognitive domains (e.g., working memory) (Whitson et al., 2018). The generalizability and reproducibility of our findings are constrained by the underrepresentation of non-white races as a result of recruitment in these segments of community-dwelling older adults, emphasizing the necessity for increased outreach efforts.

## 5 Conclusion

The present findings provide preliminary results supporting cerebrovascular function as a mechanism facilitating neuromotor resilience to cognitive loading under challenging balance conditions in older adult *APOE4* carriers who are resistant to cognitive decline. These findings extend the clinical relevance of *APOE4* as a genetic biomarker beyond the context of cognitive function and implicate its relevance in cortically-mediated balance control in motor behavioral contexts with aging. Our findings from a model of healthy aging offer the potential to create a paradigm shift in the clinical and scientific framework of balance control with aging, suggesting that exercise and physical activity interventions targeting increased cerebrovascular function may offer an effective approach to delay or prevent early clinical manifestations of cognitive interference in balance control in older adult subgroups with high genetic vulnerability to AD.

## Data Availability

The raw data supporting the conclusions of this article will be made available by the authors, without undue reservation.
